# Supplementary material for: Structural Elucidation of Antibiotic TKR2999, an Antifungal Lipodepsipeptide Isolated from the Fungus Foliophoma fallens
Source: Antibiotics (Basel). 2020 May 26;9(6):278. doi: 10.3390/antibiotics9060278 (PMC7345921; doi:10.3390/antibiotics9060278)
Supplement: Supplementary file 1 [file antibiotics-09-00278-s001.pdf]

Supporting Information

Structural Elucidation of Antibiotic TKR2999, an Antifungal Lipodepsipeptide Isolated from the Fungus *Foliophoma fallens* CF-236885

Gloria Crespo,[†] Ignacio Pérez-Victoria, [†] Francisco Javier Ortiz-López, Víctor González-Menéndez, Mercedes de la Cruz, Bastien Cautain, Pilar Sánchez, Francisca Vicente, Olga Genilloud, and Fernando Reyes*

Fundación MEDINA, Centro de Excelencia en Investigación de Medicamentos Innovadores en Andalucía, Avenida del Conocimiento 34, Parque Tecnológico de Ciencias de la Salud, E-18016 Granada, Spain

Corresponding Author
* E-mail: fernando.reyes@medinaandalucia.esTel: +34 958 993965. Fax: +34 958 846710.

Supporting Information Contents

Figure S1. ^{1}H NMR spectrum (DMSO-d_6, 24 ºC, 500 MHz) of compound **1**.
Figure S2. ^{13}C NMR spectrum (DMSO-d_6, 24 ºC, 125 MHz) of compound **1**.
Figure S3. COSY spectrum of compound **1**.
Figure S4. HSQC spectrum of compound **1**.
Figure S5. HMBC spectrum of compound **1**.
Figure S6. HSQC-TOCSY spectrum of compound **1**.
Figure S7. NOESY spectrum of compound **1**.
Figure S8. ^{1}H NMR spectrum (DMSO-d_6, 50 ºC, 500 MHz) of compound **1**.
Figure S9. Expansions of ^{1}H NMR spectrum (DMSO-d_6, 50 ºC, 500 MHz) of compound **1**.
Figure S10. JRES spectrum (50 ºC) of compound **1**.
Figure S11. Expansion of JRES spectrum (50 ºC) of compound **1** and traces of multiplets corresponding to H-2_a and H-2_b overlaid with the ^{1}H NMR spectrum.
Figure S12. Expansion of the *J*-HMBC spectrum of compound **1** (50 ºC) employed for measuring $^{3}J_{CH}$ between H-3 (fatty acid chain) and the carbon of the methyl substituent at C-4.
Figure S13. ROESY spectrum (50 ºC) of compound **1**.
Figure S14. ESI-TOF spectrum of compound **1**.
Figure S15. UV/vis (DAD) spectrum of compound **1**.
Figure S16. UV chromatogram of derivatized (L-FDVA) amino acid standards.
Figure S17. UV chromatogram of derivatized (L-FDVA) compound **1** hydrolyzate.
Figure S18. Key NOESY/ROESY correlations (dashed red arrows) and JBCA analysis employed to discard the erythro relative stereochemistry represented by the B-1/B-3 pair of rotamers.

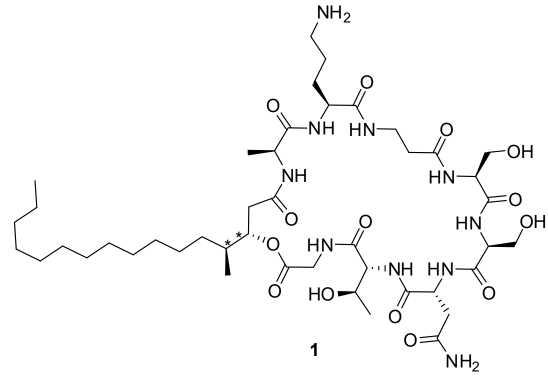

1

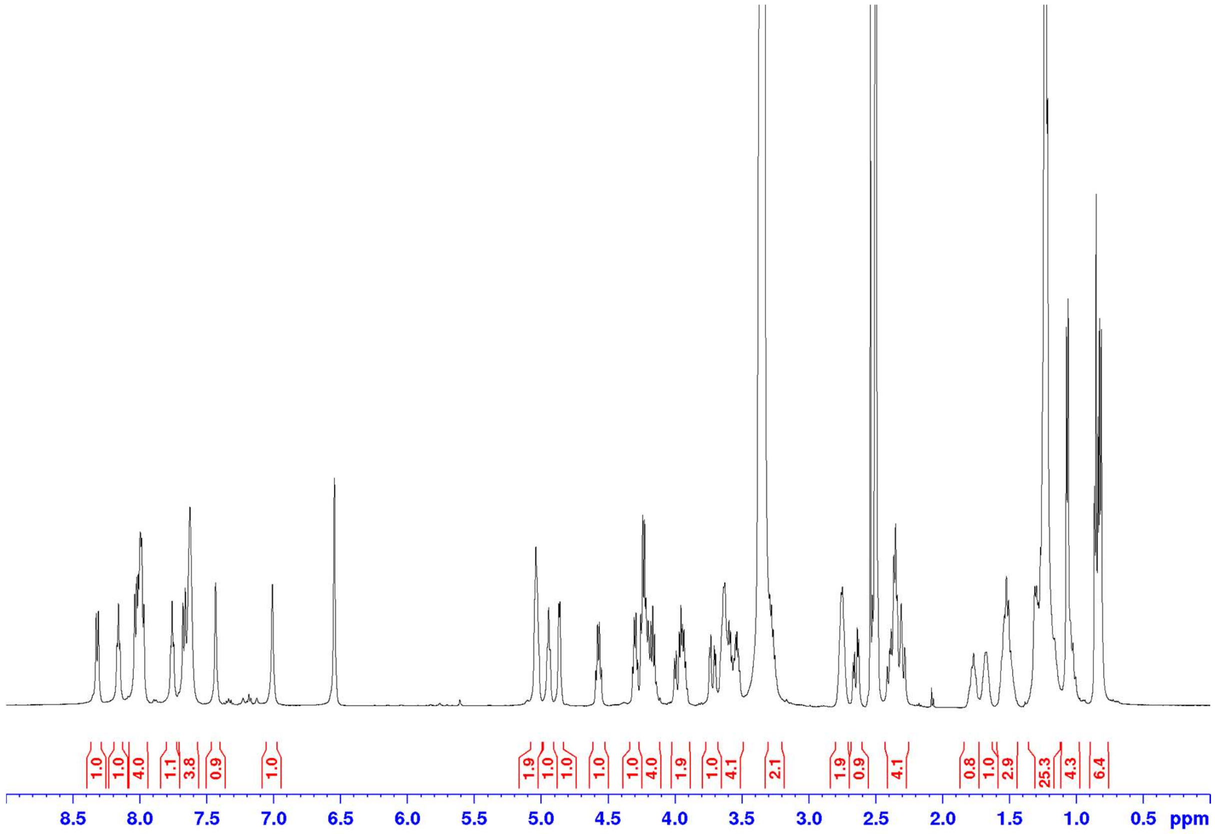

Figure S1. ^{1}H NMR spectrum (DMSO-d_6, 24ºC, 500 MHz) of compound **1**.

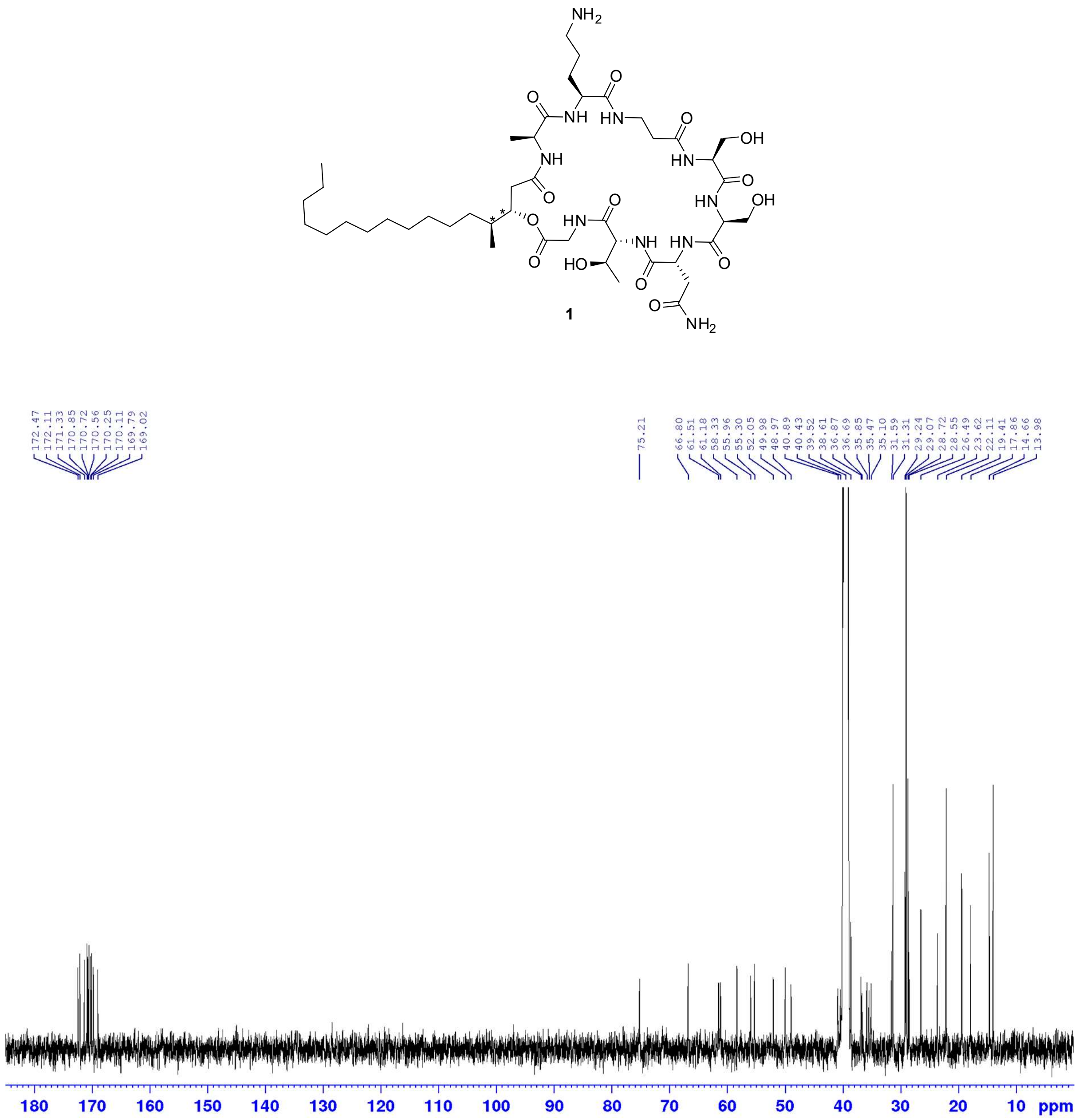

Figure S2. ^{13}C NMR spectrum (DMSO-d_6, 24ºC, 125 MHz) of compound **1**.

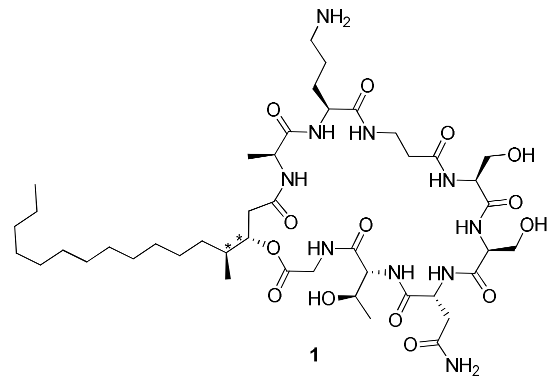

1

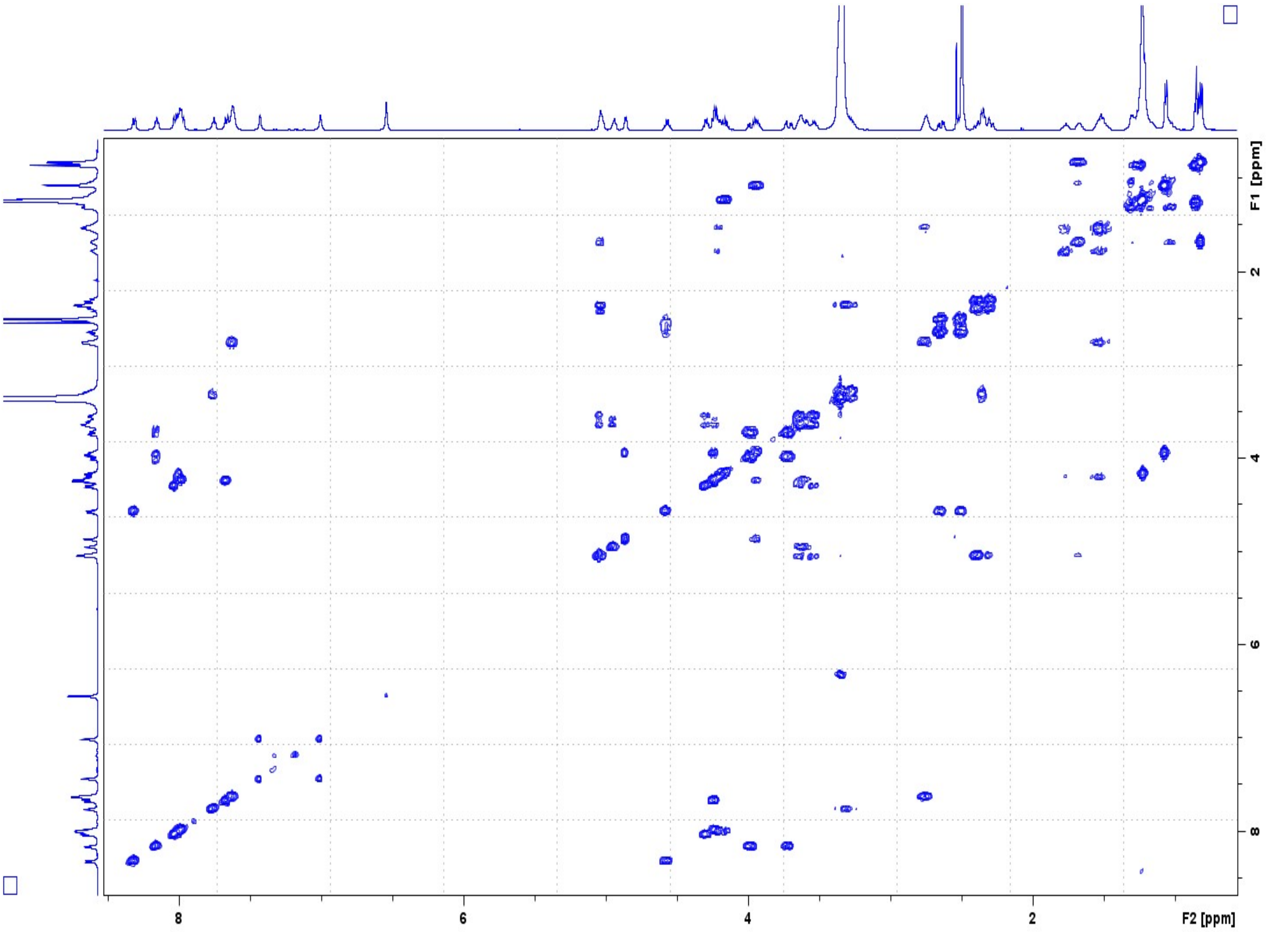

Figure S3. COSY spectrum of compound **1**.

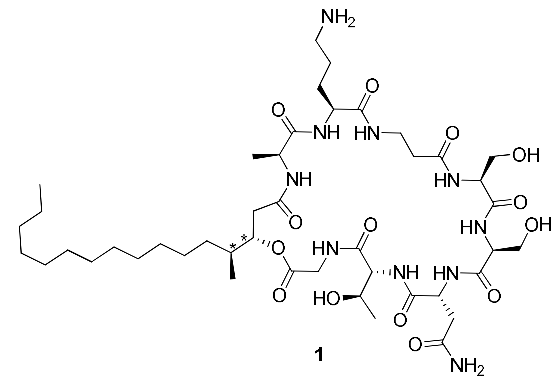

1

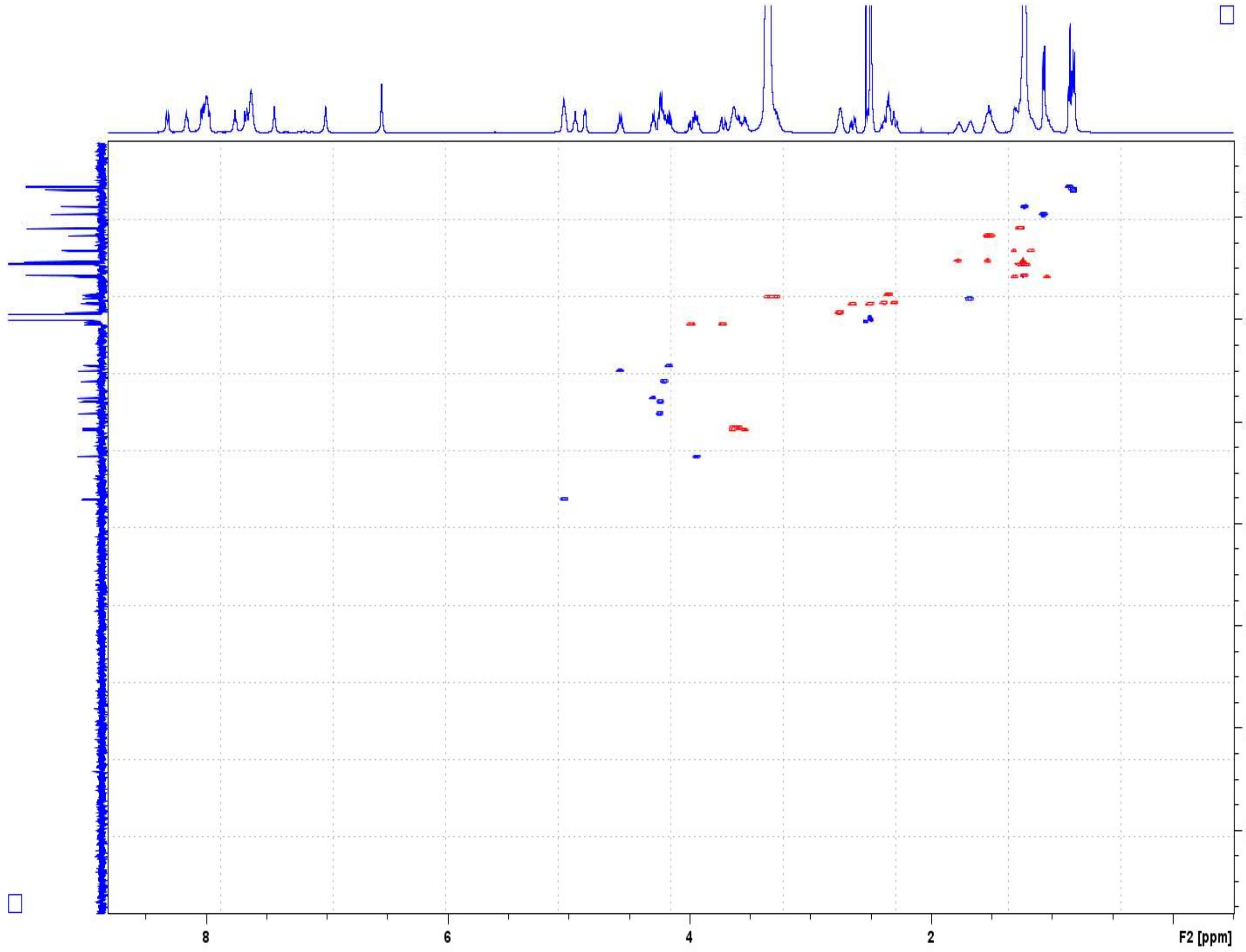

Figure S4. HSQC spectrum of compound **1**.

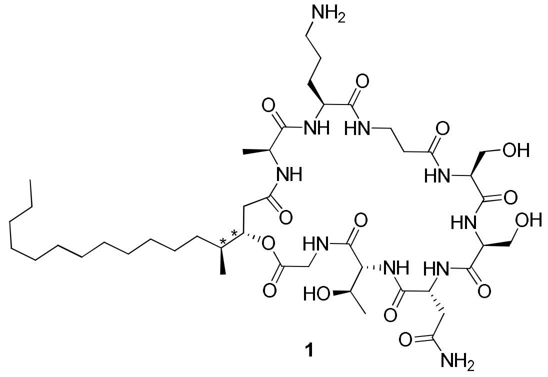

1

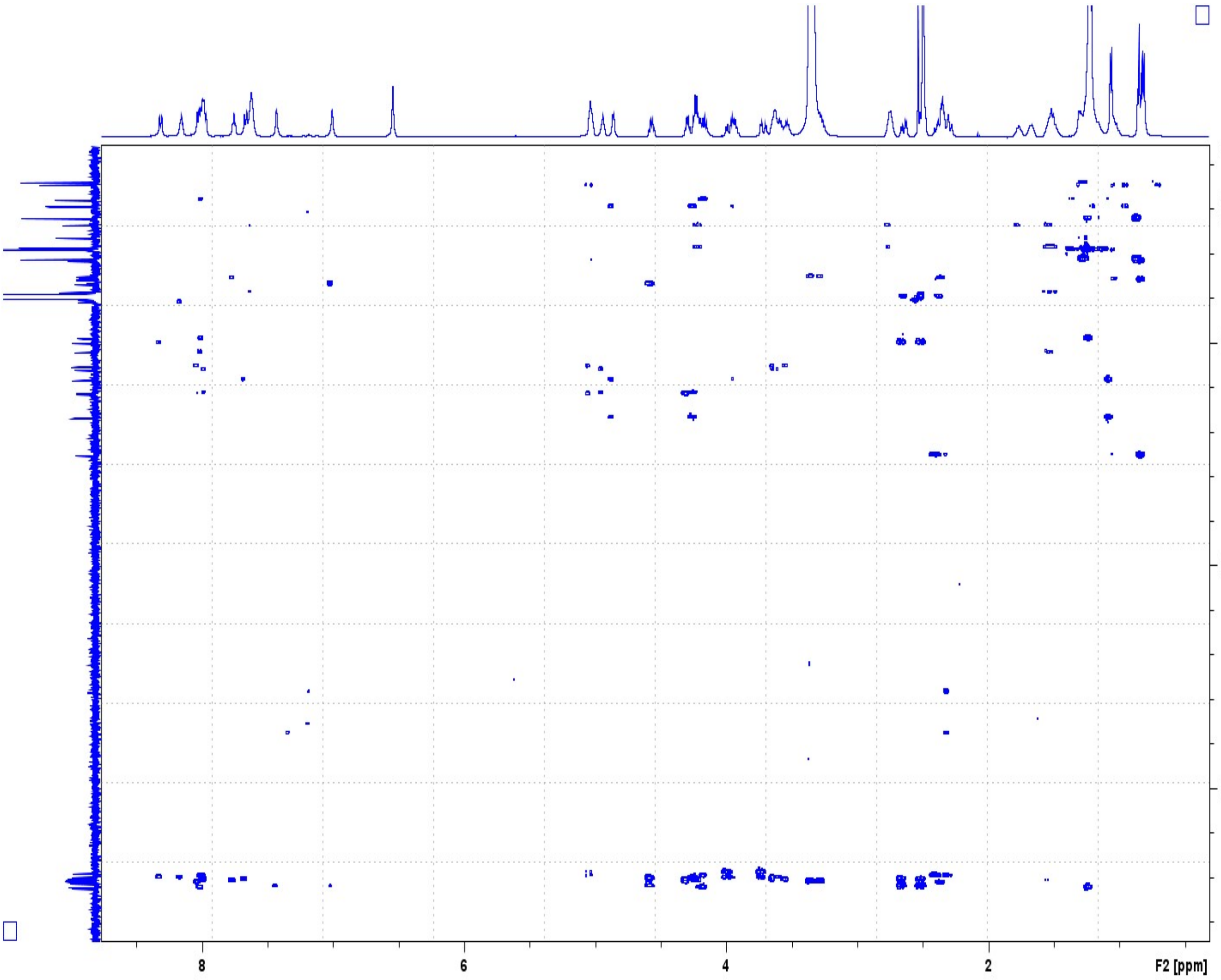

Figure S5. HMBC spectrum of compound **1**.

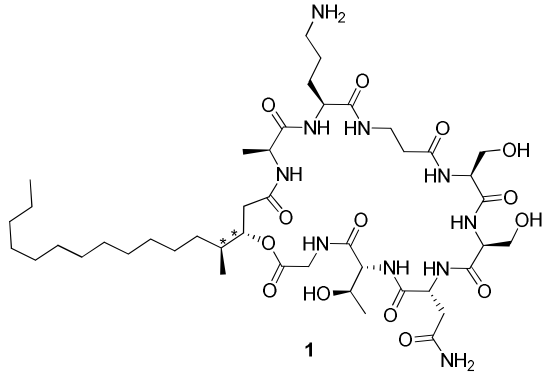

1

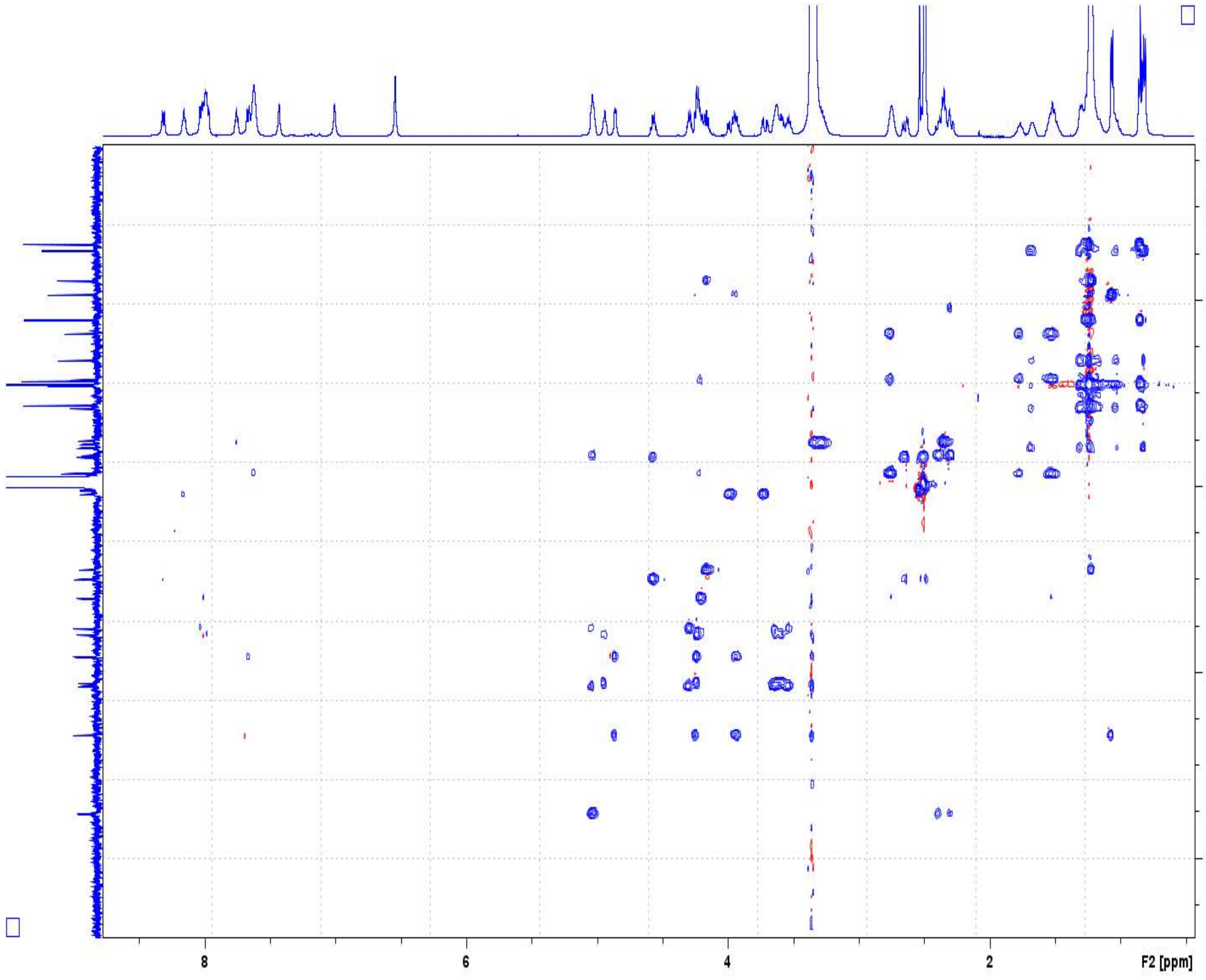

Figure S6. HSQC-TOCSY spectrum of compound **1**.

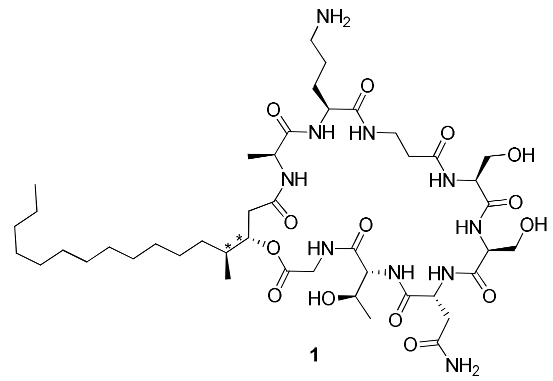

1

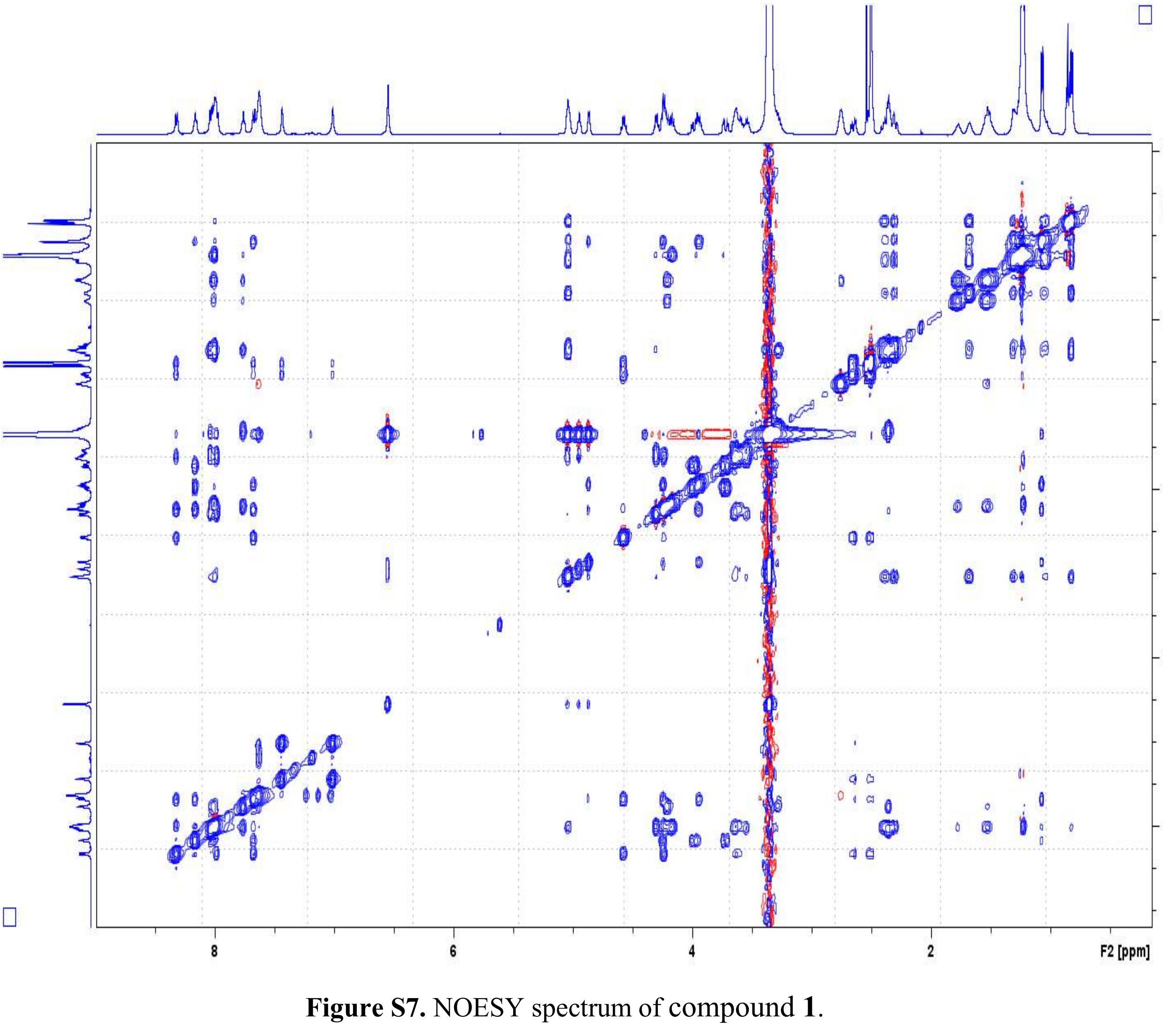

Figure S7. NOESY spectrum of compound **1**.

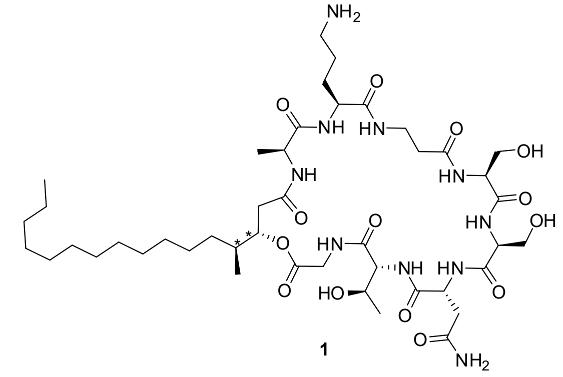

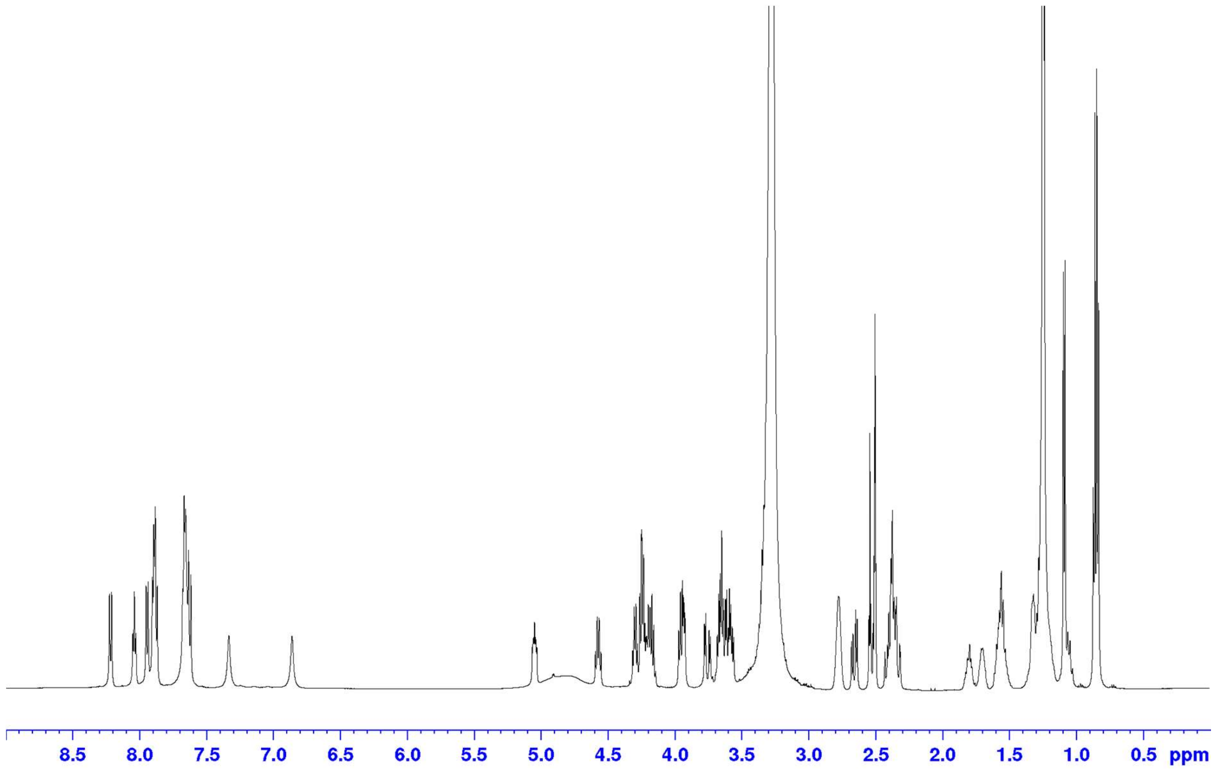

Figure S8. ^1H NMR spectrum (DMSO-d_6, 50ºC, 500 MHz) of compound **1**.

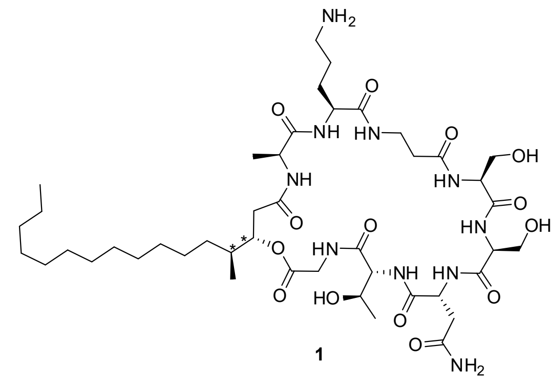

1

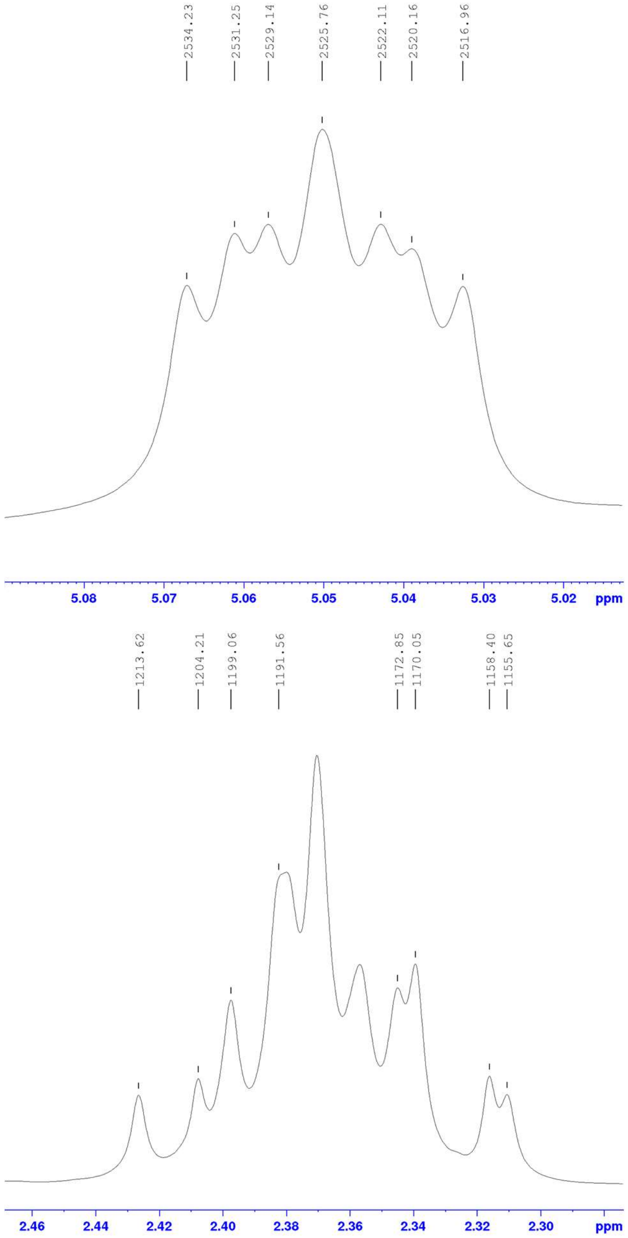

Figure S9. Expansions of ^{1}H NMR spectrum (DMSO-d$_6$, 50 ºC, 500 MHz) of compound **1**. H-3 multiplet (upper) and H-2 methylene multiplets (lower) of the fatty acid chain.

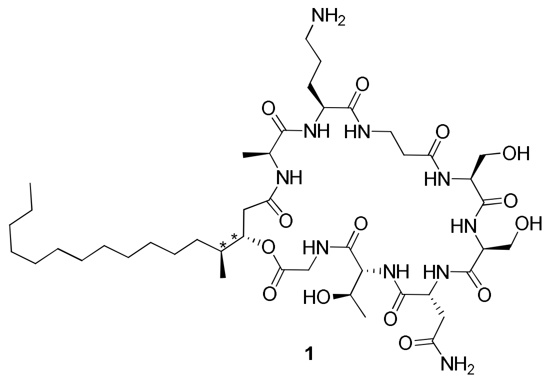

1

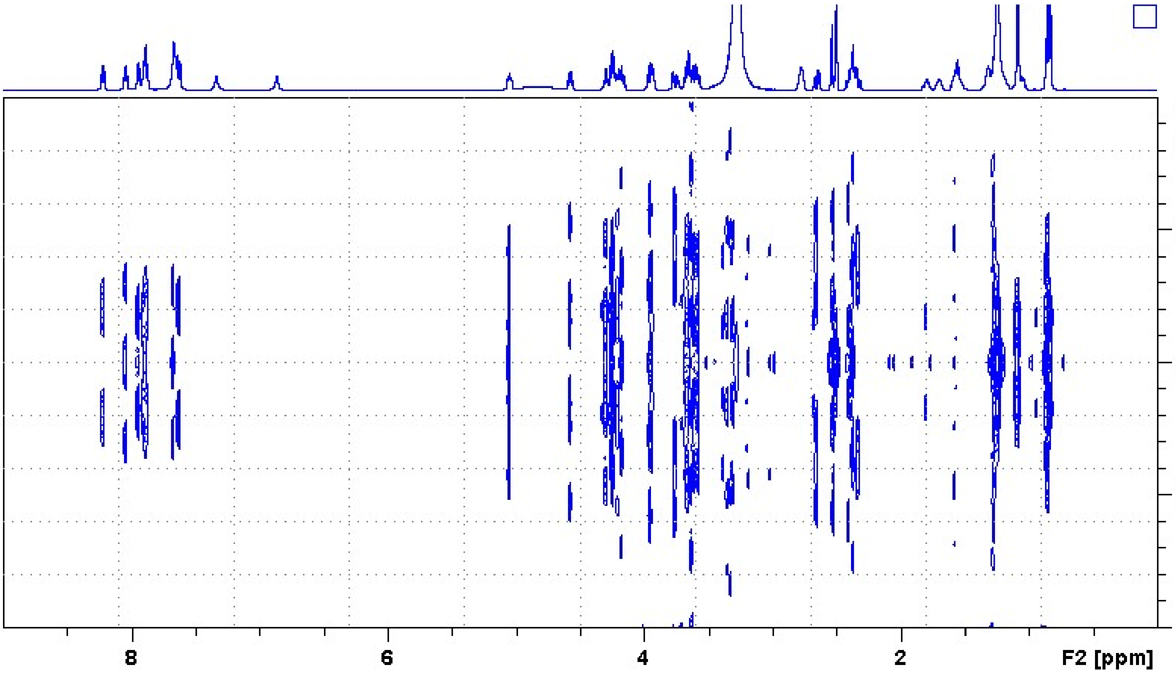

Figure S10. JRES spectrum (50 ºC) of compound **1**.

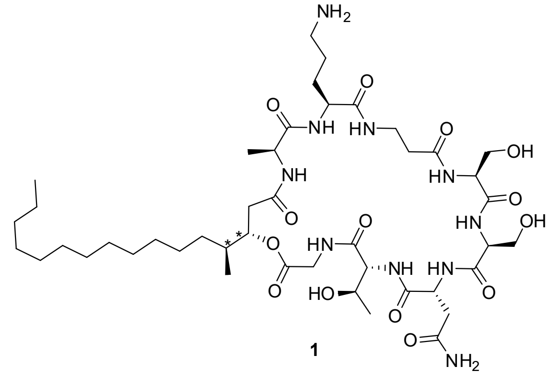

1

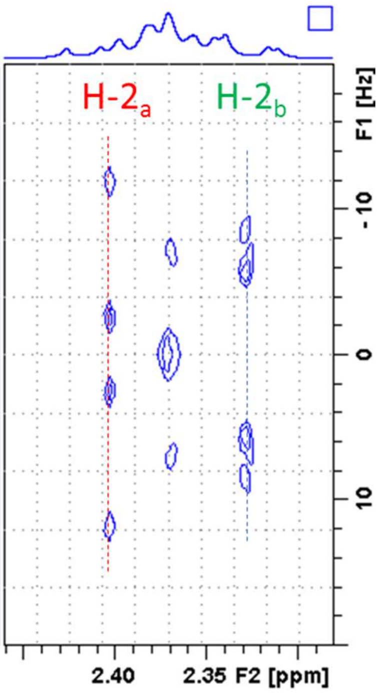

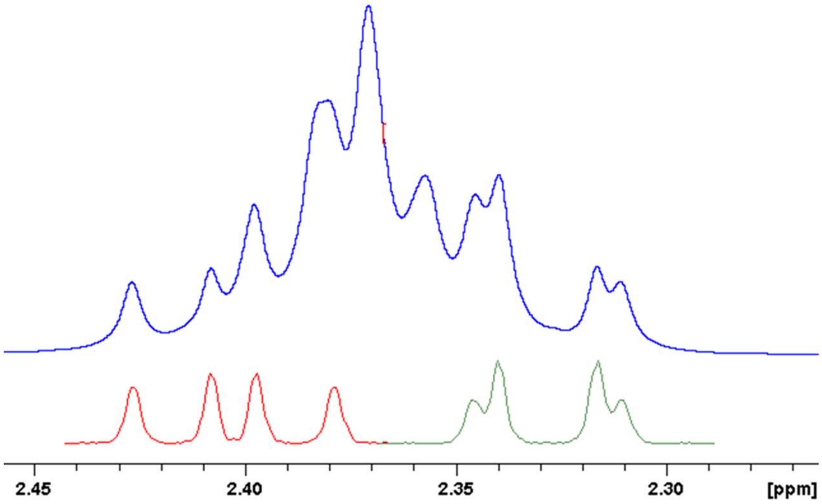

Figure S11. Expansion of JRES spectrum (50 ºC) of compound **1** and traces of multiplets corresponding to H-2$_a$ (red) and H-2$_b$ (green) overlaid with the ^{1}H NMR spectrum.

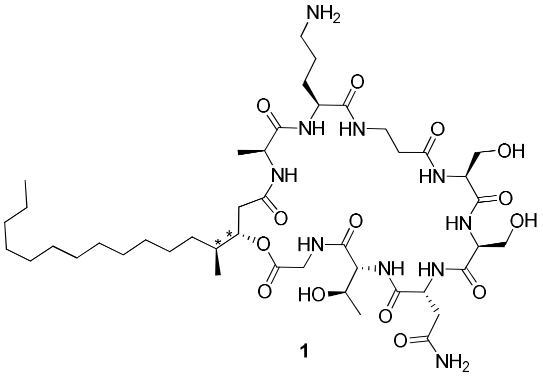

1

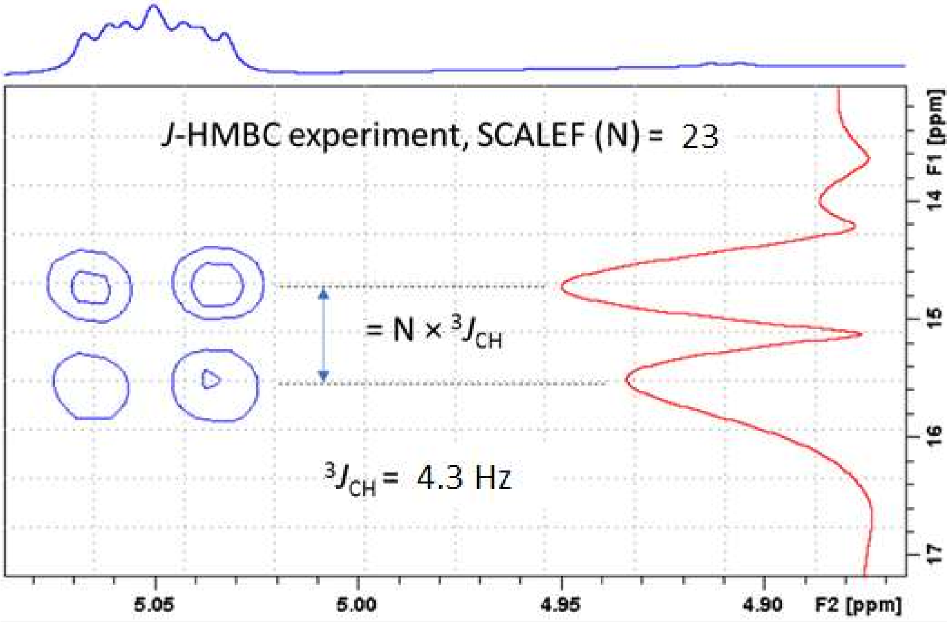

Figure S12. Expansion of the region of the *J*-HMBC spectrum of compound **1** (50ºC) employed to measure $^3J_{CH}$ between H-3 (fatty acid chain) and the carbon of the methyl substituent at C-4.

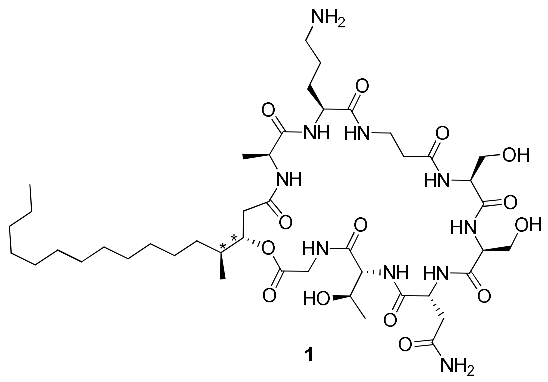

1

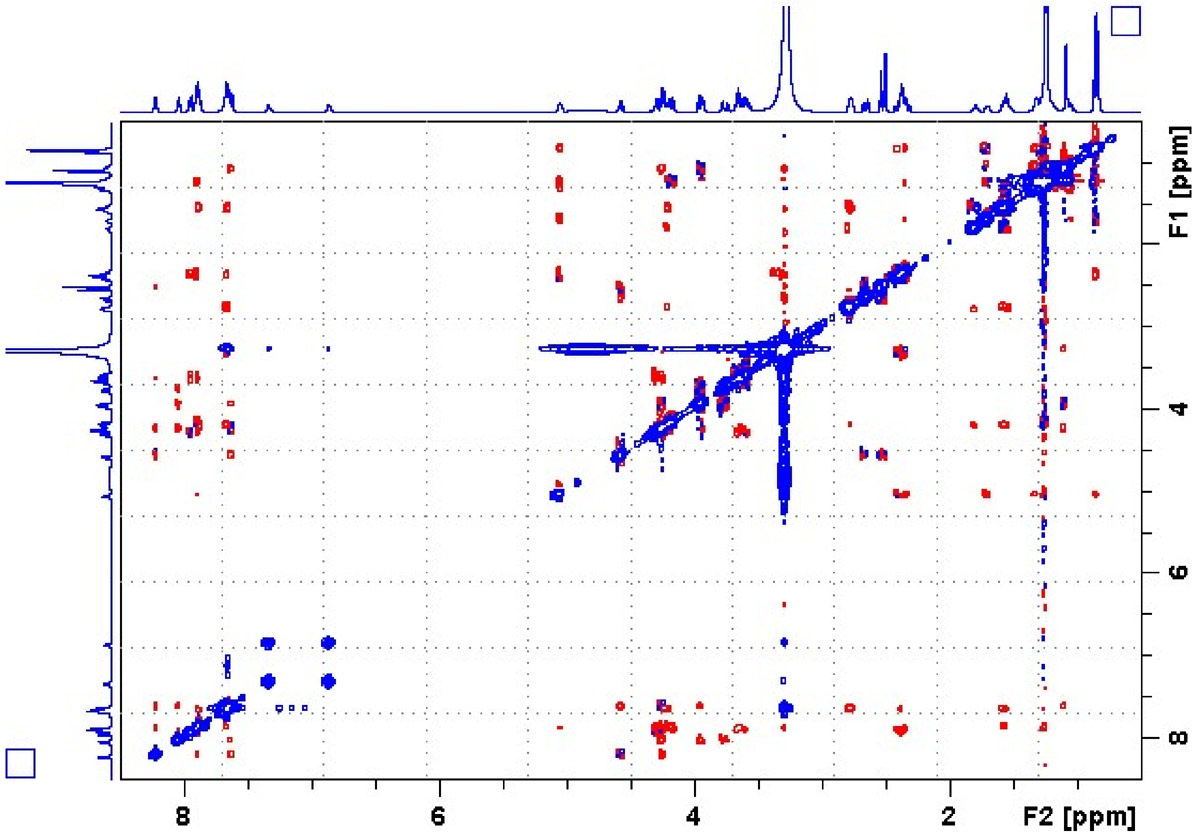

Figure S13. ROESY spectrum (50 ºC) of compound **1**.

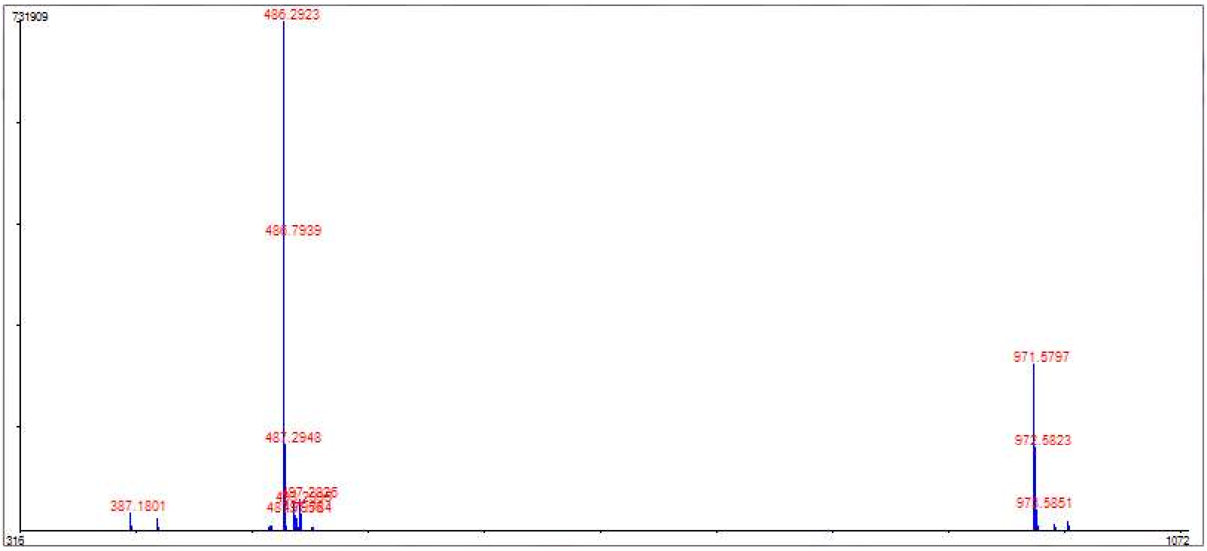

Calc. M+H$^+$= 971.5772; Error= 2.5 ppm

Figure S14. ESI-TOF spectrum of compound **1**.

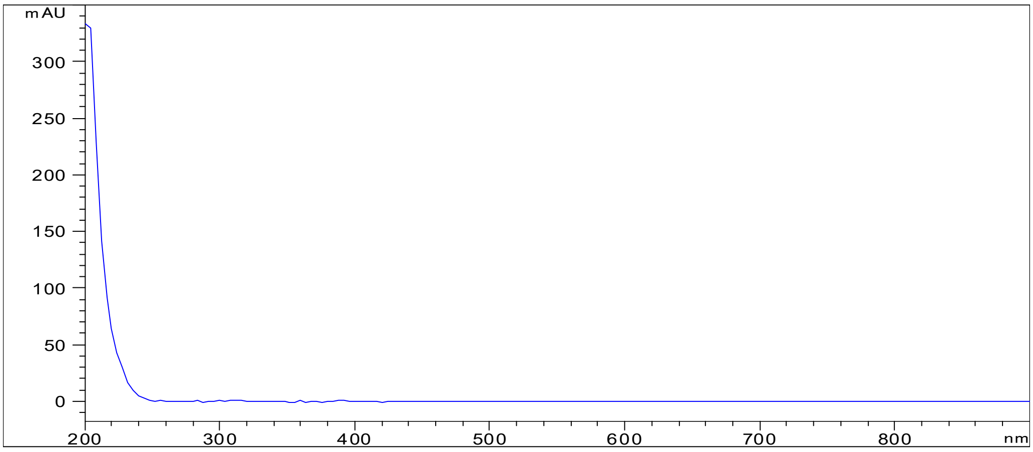

Figure S15. UV/vis (DAD) spectrum of compound **1**.

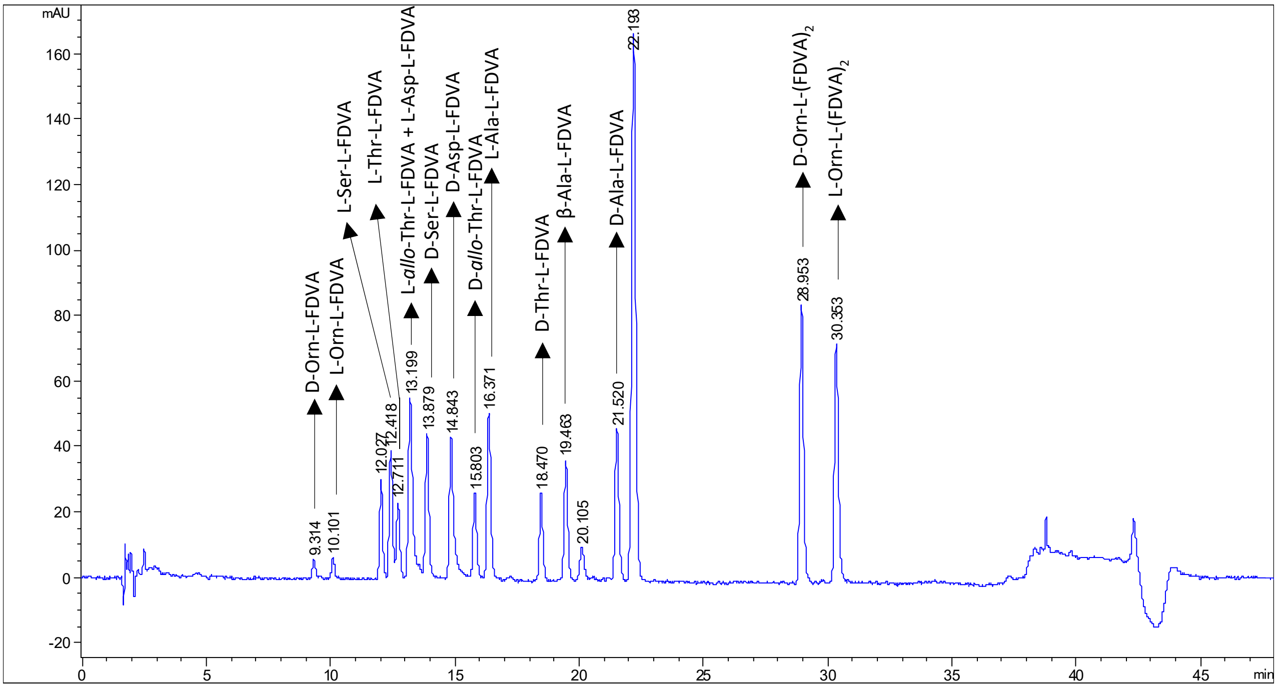

Figure S16. UV chromatogram of derivatized (L-FDVA) amino acid standards.

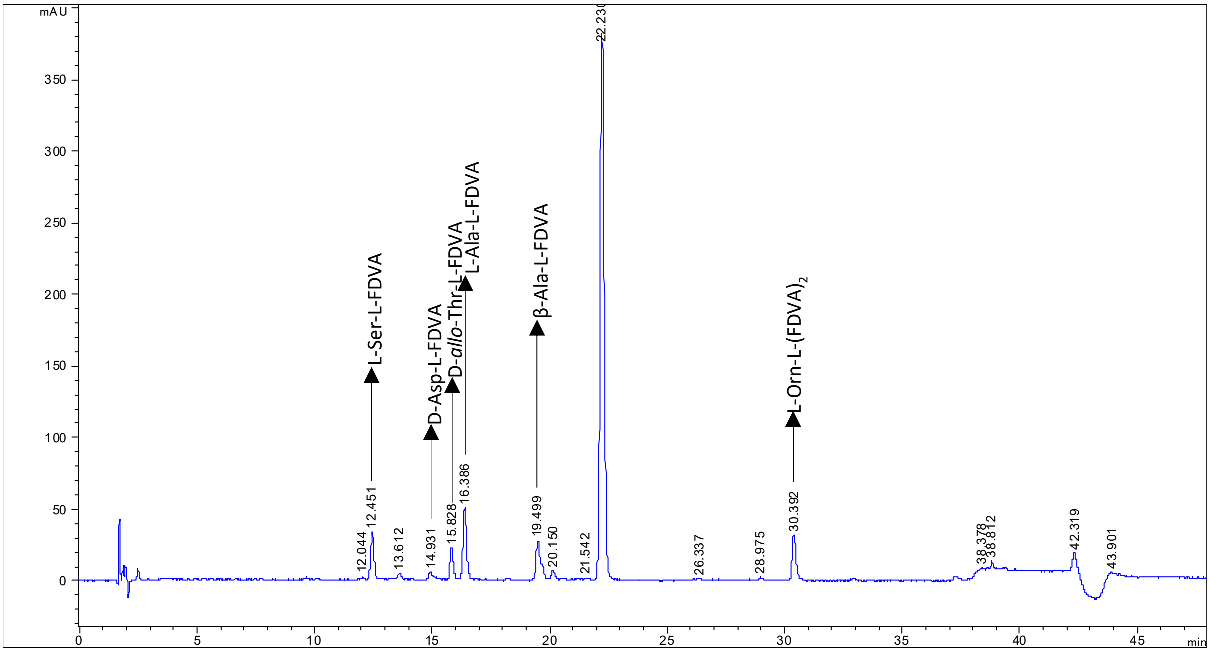

Figure S17. UV chromatogram of derivatized (L-FDVA) compound **1** hydrolyzate.

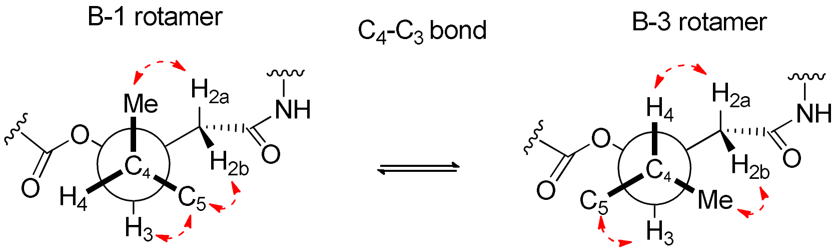

H-3/H-4, J_{HH} should be medium

H-3/Me, J_{HC} should be medium

H-4/C-2, J_{HC} should be medium but experimentally is small

Figure S18. Key NOESY/ROESY correlations (dashed red arrows) and JBCA analysis employed to discard the *erythro* relative stereochemistry represented by the B-1/B-3 pair of rotamers.
